# Proteomic analysis of ascitic extracellular vesicles describes tumour microenvironment and predicts patient survival in ovarian cancer

**DOI:** 10.1002/jev2.12420

**Published:** 2024-03-15

**Authors:** Anna Vyhlídalová Kotrbová, Kristína Gömöryová, Antónia Mikulová, Hana Plešingerová, Stanislava Sladeček, Marek Kravec, Šárka Hrachovinová, David Potěšil, Garett Dunsmore, Camille Blériot, Mathilde Bied, Jan Kotouček, Markéta Bednaříková, Jitka Hausnerová, Luboš Minář, Igor Crha, Michal Felsinger, Zbyněk Zdráhal, Florent Ginhoux, Vít Weinberger, Vitězslav Bryja, Vendula Pospíchalová

**Affiliations:** ^1^ Department of Experimental Biology, Faculty of Science Masaryk University Brno Czech Republic; ^2^ Central European Institute of Technology Masaryk University Brno Czech Republic; ^3^ Institut Gustave Roussy, INSERM U1015 Villejuif France; ^4^ Institut Necker Enfants Malades, IMMEDIAB Paris France; ^5^ Department of Pharmacology and Toxicology Veterinary Research Institute Brno Czech Republic; ^6^ Department of Internal Medicine ‐ Hematology & Oncology, University Hospital Brno and Medical Faculty Masaryk University Brno Czech Republic; ^7^ Department of Pathology, University Hospital Brno and Medical Faculty Masaryk University Brno Czech Republic; ^8^ Department of Obstetrics and Gynecology, University Hospital Brno and Medical Faculty Masaryk University Brno Czech Republic; ^9^ Department of Health Sciences, Faculty of Medicine Masaryk University Brno Czech Republic

**Keywords:** ascites, extracellular vesicles (EV), fallopian tube and peritoneum (HGSC), high‐grade serous carcinoma of the ovary, macrophage, ovarian cancer (OC), tandem mass spectrometry (MS/MS), tumour microenvironment (TME)

## Abstract

High‐grade serous carcinoma of the ovary, fallopian tube and peritoneum (HGSC), the most common type of ovarian cancer, ranks among the deadliest malignancies. Many HGSC patients have excess fluid in the peritoneum called ascites. Ascites is a tumour microenvironment (TME) containing various cells, proteins and extracellular vesicles (EVs). We isolated EVs from patients’ ascites by orthogonal methods and analyzed them by mass spectrometry. We identified not only a set of ‘core ascitic EV‐associated proteins’ but also defined their subset unique to HGSC ascites. Using single‐cell RNA sequencing data, we mapped the origin of HGSC‐specific EVs to different types of cells present in ascites. Surprisingly, EVs did not come predominantly from tumour cells but from non‐malignant cell types such as macrophages and fibroblasts. Flow cytometry of ascitic cells in combination with analysis of EV protein composition in matched samples showed that analysis of cell type‐specific EV markers in HGSC has more substantial prognostic potential than analysis of ascitic cells. To conclude, we provide evidence that proteomic analysis of EVs can define the cellular composition of HGSC TME. This finding opens numerous avenues both for a better understanding of EV's role in tumour promotion/prevention and for improved HGSC diagnostics.

## Graphical abstract

We performed the first in‐depth proteomics study of precisely isolated EVs from ascites of HGSC patients and identified core ascitic EV‐associated proteins as well as HGSC‐specific EV‐associated proteins. We provide evidence that by the combination of EV proteomics and single‐cell methods, we can assess the contribution of cells forming the tumour and its environment to EV pool in the ascites. Surprisingly, analysis of cell‐type specific EV markers showed a stronger predictive potential for HGSC patient survival than analysis of ascitic cells.

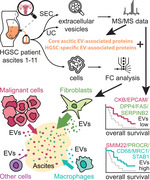

## 1 INTRODUCTION

Ovarian cancer (OC) is the eighth leading cancer in women, and even though it ranks third among gynecologic malignancies, it has the highest mortality of all gynecologic cancers, with over 200,000 deaths every year (Sung et al., 2021). The most common form of OC—high‐grade serous carcinomas (HGSC) of ovaries, fallopian tubes, and peritoneum—is responsible for 70% of OC deaths. Early stages of this disease are primarily asymptomatic but progress quickly, resulting in diagnosis at later stages (III‐IV) when metastases have already developed. As a result, the 5‐year survival rate remains dramatically low, at around 20% (Siegel et al., 2023). Unfortunately, initial positive responses to the combination of debulking surgery and chemotherapy are often reverted by the acquired chemotherapy resistance and subsequent relapse.

Unlike other cancers that utilize a haematopoietic route for metastatic transmission, HGSC preferentially spreads through transcoelomic dissemination facilitated by the ascitic fluid (Ford et al., 2020). Ascites, pathological accumulation of the fluid in the peritoneum, is often present in HGSC patients in large volumes and is removed as part of the therapeutic regimen. Therefore, it represents a unique and understudied source of patient material that mirrors the tumour's properties and microenvironment (Ford et al., 2020).

Recent work has shown that ascites affects tumour growth and progression by the contribution of various ascitic cell populations, proteins, lipoproteins and extracellular vesicles (EVs) (Kipps et al., 2013). As membranous particles, EVs are produced by cells and serve as conveyors of proteins, lipids and nucleic acids between cells. EVs are present in various bodily fluids, including blood and ascites. The control of EV molecular composition is paramount to modulating biological activity in the recipient cell. Despite the crucial role of EVs in understanding the biology of multiple pathologies and their enormous potential for diagnostics, much remains unknown. One of the reasons is that the precise isolation of EVs from complex biofluids remains a challenge due to their small size, heterogeneity and minor concentration compared with common contaminants, such as proteins and lipoprotein particles. Several studies have recently attempted to find an ideal EV isolation method (Azkargorta et al., 2021; Brennan et al., 2020; Dong et al., 2020; Holcar et al., 2021; Takov et al., 2019; Tian et al., 2020; Veerman et al., 2021), but it seems that such a unified method does not exist, considering the versatility of downstream applications. Additional studies have recommended sequentially combining two isolation methods based on different principles (so‐called orthogonal methods) to increase the purity of isolated EVs (Karimi et al., 2018; Vergauwen et al., 2021). However, the inherent disadvantage of this approach is its considerably lower yield because more steps in EV isolation lead to the loss of more EVs in the process (Brennan et al., 2020; Wei et al., 2020).

In this study, we comprehensively describe the protein composition of EVs from ascites of HGSC patients. In parallel we isolated EVs by a variant of differential ultracentrifugation and size‐exclusion chromatography and analyzed these EV‐rich fractions by tandem mass spectrometry (MS/MS). We have defined the list of core ascitic EV‐associated proteins that can be a reference for future studies. Further, the subtraction of proteins detected in EVs from benign ovary/peritoneum‐related fluids allowed us to define HGSC‐specific EV‐associated proteins that represent candidate EV‐associated HGSC biomarkers. Finally, taking advantage of the availability of single‐cell RNA sequencing (scRNA seq) data of cellular components of the HGSC ascites, we have identified the prospective cellular sources of ascitic EVs (mainly malignant cells, fibroblasts and macrophages) and their differential contribution to the EV pool in each analyzed HGSC ascites. EV analysis thus represents a valuable and comprehensive way to describe the key features of cellular TME in HGSC. As such, it holds the potential to not only describe the complex states of HGSC tumours and their microenvironment in individual patients but also open multiple avenues for personalized medicine of HGSC.

## 2 MATERIALS AND METHODS

### 2.1 Patient samples and ethics statement

The cohort was selected from patients undergoing treatment at the Department of Obstetrics and Gynecology of University Hospital Brno, Czech Republic. Written informed consent approved by the Ethics Committee of University Hospital Brno (reg. number: 02–100719EK) was obtained from each concerned patient. The study was approved by the Ethics Committee of Masaryk University (reg. numbers: MUNI/M/1050/2013, 17–11776Y and 0229/2019) and the Ethics Committee of University Hospital Brno (reg. number: 02–100719EK) and was conducted in accordance with the Declaration of Helsinki. All specimens were handled according to ethical and legal standards. All samples were reviewed and classified according to the current WHO classification of tumours. Pseudonymized clinicopathological data accompanied every clinical specimen. Ascites were collected by oncogynaecologists during primary cytoreductive surgery of patients diagnosed with HGSC. Non‐malignant ascites from ovarian hyperstimulation syndrome or follicular fluid of women undergoing in vitro fertilization (IVF) treatment were used as controls. Patient characteristics are available in Table S1.

Ascites/control fluid was collected into sterile bottle/falcon tube and transported to the laboratory at 4°C within few hours after collection from patient. Cells and cell debris were depleted from the fluid by centrifugation at 200 × *g* and 1500 × *g*, respectively. Cell‐ and debris‐free ascites/control fluids were stored in deep freezer (−80°C) until used for isolation of EVs.

### 2.2 Ultracentrifugation

Isolation of EVs by differential ultracentrifugation coupled to sucrose cushion flotation step was performed as described previously (Kotrbová et al., 2019; Pospichalova et al., 2015). Briefly, cell‐ and debris‐free ascites/control fluid/conditioned medium from Caov‐3 and Kuramochi cell lines (cultured with 10% EV‐free FBS) was centrifuged in 38‐mL thinwall polyallomer UC tubes using SW32Ti rotor and Optima XPN 90k ultracentrifuge (all Beckman Coulter) at 14,000 × *g*, 1:10 h, 4°C. The supernatant was then transferred into new tubes and ultracentrifuged at 100,000 × *g*, 3:10 h, 4°C. Pellet from this ultracentrifugation was resuspended in filtered PBS (fPBS; using 0.22 µm PVDF filter) and layered onto 4 mL of sucrose cushion (30% sucrose in 20 mM Tris pH 7.6 in D_2_O, filter sterilized) in a new tube and ultracentrifuged at 100,000 × *g*, 1:10 h, 4°C. Approximately 6 mL of fPBS and cushion at the interface of both layers was aspirated to ensure that all EVs were collected, transferred into a new tube, tube was filled with fPBS and ultracentrifuged at 100,000 × *g*, 1:10 h, 4°C. The supernatant was discarded and pellet of EVs was resuspended in approximately 100 µL of fPBS. This fraction was further referred to as U.

### 2.3 Size‐exclusion chromatography

Cell‐ and debris‐free ascites was concentrated 2.5 times to 1 mL using Pierce Protein Concentrator PES, 10K MWCO (88517, Thermo Scientific). qEV Original (70 nm) (IZON Science LTD) size‐exclusion chromatography column was used according to manufacturer's instructions. 1 mL of concentrated ascites was loaded onto the column and followed by elution of 24 sequential 0.5 mL eluate fractions. Fractions 8–9 containing the highest peak of EVs were pooled and further referred to as S fraction and fractions 14–15 containing the highest concentrations of protein were pooled and further referred to as B fraction.

### 2.4 Cryo‐electron microscopy

Samples for cryo‐EM were vitrified using FEI vitrobot Mark IV. 3.5 µL of sample was applied on freshly plasma‐cleaned Cu (Quantifoil R 2/1 200 or 300 mesh) grids and vitrification in liquid ethane was performed with following settings: blot force −4; blot time 6 s; wait time 120 s; 100 % humidity at 4°C. The grids were subsequently mounted to the Autogrid cartridges and imaged on Talos Arctica (ThermoFisher Scientific) cryo‐TEM microscope operated at 200 kV using Falcon3 direct electron detection camera at 5300−8500 × (for overview) or 73,000−92,000 × (for detail) nominal magnification with the underfocus in the range 3−10 µm and the overall dose of <20 e/Å2.

### 2.5 Dynamic light scattering

Size and concentration characterization was done with Multi‐angled dynamic light scattering technique (MADLS®). Approximately 50 µL of the sample suspension was placed in low volume quartz batch cuvette ZEN2112 (Malvern Panalytical Ltd, UK) and measured using Zetasizer Ultra (Malvern Panalytical Ltd, UK) equipped with HeNe Laser (633 nm) and three detection angles: 173°, 90° and 13°. The measurements were performed at a constant temperature of 25°C. Obtained data were evaluated using ZS Xplorer software version 1.5.0.163 (Malvern Panalytical Ltd, UK).

### 2.6 SDS‐PAGE and western blotting

Protein sample lysates were prepared by direct lysis of EVs/fraction isolates in 5× concentrated reducing Laemmli buffer. Notably, samples designated for the detection of TACSTD2, MRC1 and FCGR1A were lysed in non‐reducing Laemmli buffer to preserve specific interactions Lysates were then separated according to their molecular mass on 15% SDS‐PAGE and transferred to Immobilon‐P Membrane (Millipore). Membranes were blocked in 5% non‐fat milk in PBS with 0.5% Tween 20 and then incubated with primary antibodies overnight at 4°C. Next step was extensive washing in PBS with 0.5% Tween 20, followed by incubation with secondary antibodies. After final extensive washing, membranes were overlayered by chemiluminescent ECL solutions Immobilon Western (Millipore) and the signal was detected using Fusion SL (Vilber Lourmat). The following antibodies were used: rabbit anti Flotillin1 (A3023, Exbio, dilution 1:500), mouse anti Flotillin2 (BD610383, BD, dilution 1:500), mouse anti CD81 (105601, Caprico Biotechnologies, dilution 1:500), rabbit anti CD9 (A19027, ABclonal, dilution 1:500), rabbit anti ApoA‐I (sc‐30089, Santa Cruz Biotechnology, dilution 1:1,000), rabbit anti FAS (A21903, ABclonal, dilution 1:1,000), mouse anti TACSTD2 (TM0051, ECM Biosciences, dilution 1:500), mouse anti CD68 (BD556078, BD, dilution 1:500), goat anti MRC1 (sc‐34577, Santa Cruz Biotechnology, dilution 1:500), mouse anti FCGR1A (sc‐1184, Santa Cruz Biotechnology, dilution 1:500).

### 2.7 Amido black staining

The blotting membrane was incubated for 5 min in amido black staining solution [0.1% (w/v) amido black dissolved in 4% (v/v) ethanol and 1% (v/v) glacial acetic acid] and then washed in destaining solution for 3 × 5 min [(40% (v/v) ethanol, 10% (v/v) glacial acetic acid and 2% (v/v) glycerol]. Finally, the membrane was rinsed in dH_2_O, air‐dried and scanned using Fusion SL (Vilber Lourmat).

### 2.8 Samples preparation for LC‐MS/MS analyses

Individual samples for liquid chromatography (LC) coupled to tandem mass spectrometry (MS/MS) were mixed with 5% SDS, 250 mM DTT, 250 mM TrisHCl buffer (sample:buffer ratio 4:1) and incubated for 30 min at 95°C. Resulting protein solutions were processed by filter‐aided sample preparation (FASP) method (Wiśniewski et al., 2011) with some modifications. The samples were mixed with 8 M UA buffer (8 M urea in 100 mM Tris‐HCl, pH 8.5), loaded onto the Microcon device with MWCO 30 kDa (Merck Millipore) and centrifuged at 7000 × *g* for 30 min at 20°C. The retained proteins were washed (all centrifugation steps after sample loading were done at 14,000 × *g*) with 200 µL UA buffer. The final protein concentrates kept in the Microcon device were mixed with 100 µL of UA buffer containing 50 mM iodoacetamide and incubated in the dark for 20 min. After the next centrifugation step, the samples were washed three times with 100 µL UA buffer and three times with 100 µL of 50 mM NaHCO_3_. Trypsin (500 ng; sequencing grade, Promega) was added onto the filter and the mixture was incubated for 18 h at 37°C. The tryptic peptides were finally eluted by centrifugation followed by two additional elutions with 50 µL of 50 mM NaHCO_3_. Peptides were directly after FASP extracted into LC‐MS vials by 2.5% formic acid (FA) in 50% acetonitrile (ACN) and 100% ACN with addition of polyethylene glycol (20,000; final concentration 0.001%) (Stejskal et al., 2013) and concentrated in a SpeedVac concentrator (Thermo Fisher Scientific) prior LC‐MS/MS analyses.

### 2.9 LC‐MS/MS analysis of peptides

LC‐MS/MS analyses of all peptide mixtures were done using nanoElute system (Bruker) connected to timsTOF Pro mass spectrometer (Bruker). Two column (trapping column: Acclaim™ PepMap™ 100 C18, dimensions 300 µm ID, 5 mm long, 5 µm particles, Thermo Fisher Scientific; separation column: Aurora C18, 75 µm ID, 250 mm long, 1.6 µm particles, Ion Opticks) mode was used on nanoElute system with default equilibration conditions (trap column: 10 volumes at 217.5 bars; separation column: 4 column volumes at 800 bars). Sample loading was done using 3 pickup volumes +2 µL at 100 bars. Trapped peptides were eluted by 60 min linear gradient program (flow rate 400 nL/min, 2%–30% of mobile phase B; mobile phase A: 0.1% FA in water; mobile phase B: 0.1% FA in acetonitrile) followed by system wash step at 80% mobile phase B. The analytical column was placed inside the Column Toaster (40°C; Bruker) and its emitter side was installed into CaptiveSpray ion source (Bruker).

MS data were acquired in m/z range of 100–1700 and 1/k0 range of 0.6‐1.6 V×s×cm^−2^ using DDA‐PASEF method acquiring 10 PASEF scans with scheduled target intensity of 20,000 and intensity threshold of 2500. Active exclusion was set for 0.4 min with precursor reconsideration for 4× more intense precursors. For more details on the method used, please inspect the raw data.

For data evaluation, we used MaxQuant software (v1.6.17) (Cox & Mann, 2008) with integrated Andromeda search engine (Cox et al., 2011). Search was done against protein databases of UniProtKB Human (20,609 protein sequences, version from 2020‐12‐02, downloaded from https://ftp.uniprot.org/pub/databases/uniprot/current_release/knowledgebase/reference_proteomes/Eukaryota/UP000005640/UP000005640_9606.fasta.gz) and cRAP contaminants (112 sequences, version from 2018‐11‐22, downloaded from http://www.thegpm.org/crap). Modifications were set as follows for database search: oxidation (M), deamidation (N, Q) and acetylation (Protein N‐term) as variable modifications, with carbamidomethylation (C) as a fixed modification. Enzyme specificity was tryptic/P with two permissible missed cleavages. Only peptides and proteins with false discovery rate threshold under 0.01 were considered. Match between runs was switched on with the default settings used except match time window (0.5 min).

### 2.10 Bioinformatic analysis

ProteinGroups.txt file, the output of MaxQuant, was further processed using R (version 4.3.0). Common contaminants (cRAP proteins, and proteins containing ‘keratin/Keratin’ in Fasta headers), Reverse sequences and Only Identified by Site were removed. Gene names were updated using HGNChelper package (v. 0.8.1, against version from 2022‐08‐16) in our proteomic dataset as well as in all publicly available datasets used in this study. Except for Figures 2, 6, 6b’, 6b’’, 7e, 7e’, 7f, 7f’ and 8b binary information based on intensity (intensity > 0 means protein was detected, intensity = 0 means protein was not detected) was used. In Figure 6a, a’ the cell type‐specific markers were adopted from (Izar et al., 2020), the list was cropped to contain the same number of markers (top 28 differentially expressed) for each cell type. In Figure 6b, b’, relative contribution was calculated for each patient as (mean intensity per cell type)/(sum of intensities per patient) *100. Table of MISEV markers was generated based on MISEV2018 (Théry et al., 2018). Heatmaps were created using ComplexHeatmap package (v. 2.14.0). Upset plots were generated using UpSetR package (v. 1.4.0). For gene ontology analysis (biological process, cellular compartment), we employed gProfiler2 package (v. 0.2.1, version of database e109_eg56_p17_1d3191d). The Pathway Analysis was performed against Reactome version 86 on 11/12/2023. Data for Figure S1c were adopted from (Lischnig et al., 2022). Small EV proteins were categorized based on threshold *p*‐value < 0.05 and Fold Change (sEV/lEV) > 1.5 and large EV proteins were identified using a *p*‐value < 0.05 and Fold Change (sEV/lEV) <−1.5, others were considered ‘nonsignificant’. The proteins detected in our data but absent from the referenced study (Lischnig et al., 2022) are labeled as ‘not detected’. Additionally, for assignment of cellular localization we employed the Human Cell Map resource (Go et al., 2021). To analyze the cell type markers proportions, we used the CellKB database (Patil & Patil, 2022) and data from Izar et al. (2020). Cell types of EVs isolated by SEC and UC were compared by repeated measure correlation (Bakdash & Marusich, 2017) using rmcorr R package (v. 0.5.4). Fold enrichment was computed as (% of EVs)/(% of cells) and statistical analysis was conducted using repeated measures ANOVA using rstatix R package (v. 0.7.2).

### 2.11 Survival analysis

For survival analysis, 41 cell type‐specific markers adopted from (Izar et al., 2020), which were detected in EVs isolated by both methods, and additional four cell type‐specific markers identified in Figure 5b (MRC1, IDH2, FAS and ITGB8) were selected. The four remaining proteins from ‘TOP8 HGSC‐specific EV‐associated proteins’ identified in Figure 5b (CD68, LILRB1, FCGR1A and TACSTD2) are encompassed among the 41 cell type‐specific markers adopted from (Izar et al., 2020). Our selection criteria were as follows: (i) protein detected by UC, as an isolation method which allows detection of rare EV‐associated proteins; (ii) cut‐off above 100,000 and (iii) cut‐offs dichotomizing the cohort into subgroups with at least three patients. Similarly, for cellular composition we analyzed the proportions of cell types derived from FC data and selected cut‐offs dichotomizing the cohort into subgroups with at least three patients. Subsequently, for both analyses, all resulting cut‐offs were assessed, and for each protein/cell type, the cut‐off yielding the lowest *p*‐value was chosen. Overall survival (OS) and hazard ratios (HR) were calculated using the log‐rank test and Cox proportional hazards model, respectively, in a univariate analysis.

### 2.12 Data and code availability

We have submitted all relevant data of our experiments to the EV‐TRACK knowledgebase (EV‐TRACK ID: EV231008) (Van Deun et al., 2017).

The mass spectrometry proteomics data have been deposited to the ProteomeXchange Consortium via the PRIDE (Perez‐Riverol et al., 2022) partner repository with the dataset identifier PXD041751.

The code and additional primary data are available on Github, under DOI: https://zenodo.org/records/10396295.

### 2.13 Graphics

All schemes were created in Affinity Designer (version 1.10.5, Pantone LLC) or Illustrator (version CS6, Adobe). Survival analysis plots were generated using Prism, version 5.0, GraphPad Software.

### 2.14 Flow cytometry analysis

Ascites cells were thawed and approximately 100 µL cell pellet of each ascites was used for staining. Cells were re‐suspended in 100 µL of ‘supermix’ including LIVE/DEAD Blue (Molecular Probes™ L23105): 0.1 µL of probe/100 µL of FACS buffer (2% FBS + 0.1% sodium azide in Ca/Mg^2+^‐free PBS). Samples were then incubated in the dark, at 4°C for 20 min. After washing with FACS buffer cells were re‐suspended in 100 µL of antibody ‘mastermix’, pipetting and incubated in the dark, at 4°C for 30 min. The antibody ‘mastermix’ contained the panel of antibodies diluted 1:200 in FACS buffer as specified in Table S7. After incubation with antibodies, the samples were topped up with 1 mL of FACS buffer and spun at 300 × *g*, 5 min, 20°C. Cells were again re‐suspended in 200 µL of FACS buffer and the whole suspension was filtered through 70 µm cell‐strainer cap (352235, Falcon), vortexed and directly used for flow cytometry (FC) measurement using Cytek® Aurora spectral flow cytometer. Data processing and analysis was done in FlowJo™_v10.8.1. software and flowCore and CATALYST packages in R.

## 3 RESULTS

### 3.1 Protein composition of EVs from HGSC patients´ ascites

**FIGURE 1:**
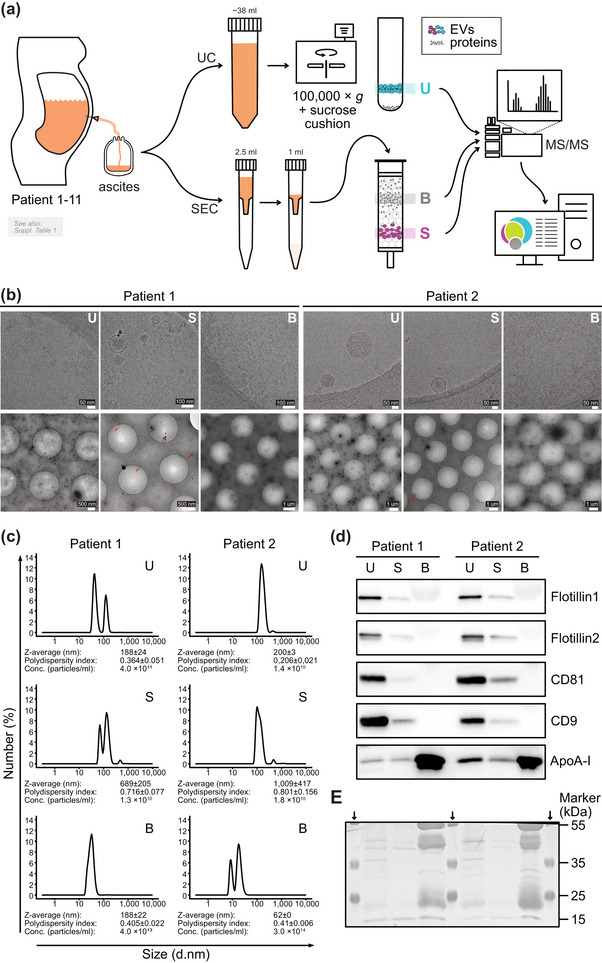
Isolation and characterization of EVs. (a) Schematic overview of processing and isolation of samples from patient ascites prior the mass spectrometry (MS) analysis. Three different samples were isolated from each patient ascites. Samples U stand for EVs isolated by differential ultracentrifugation coupled with sucrose cushion purification step; samples S and B were both obtained from size‐exclusion chromatography of concentrated ascitic fluid: S fraction contains the majority of EVs, whereas B fraction contains the bulk of proteins and served as background of contaminating proteins for each patient sample. Due to principle of each method, different input volumes of ascites were used: 38 mL for sample U and 2.5 mL (concentrated to 1 mL) for samples S and B. (b) Representative Cryo‐EM images of samples—close‐up in the top and overview in the bottom. Red arrows in S images show EVs present in the overview image (due to their low count). (c) DLS measurements of EVs. Each graph shows average of three separate measurements for the sample. Size and polydispersity index are reported as mean ± standard deviation (SD), concentration as mean (*n* = 3). (d) WB analysis for EVs markers/EV‐associated proteins and a ‘negative marker’/non‐EV associated protein ApoA‐I. (e) Amidoblack staining for total protein on the membrane. Staining was performed after WB analysis.

We collected primary ascitic biopsies from 11 patients with HGSC to analyze the protein content of the ascitic EVs. Basic patient characteristics are provided in Table S1. Given the limitations of individual EV isolation approaches, we isolated EVs using ultracentrifugation coupled to sucrose cushion step (UC) and size‐exclusion chromatography (SEC) to capture a broader range of EVs present in each sample. EVs isolated by these methods (U for UC, S for SEC), as well as the primary protein fractions from size‐exclusion chromatography (B)—serving as a negative control for each patient, were then subjected to MS/MS analysis (Figure 1a). The purification process was verified by the analysis of individual fractions using several quality‐control methods routinely applied in the EV research. Cryo‐electron microscopy (Cryo‐EM) confirmed that circular EVs with double‐layered membranes are present only in EV‐containing fractions U and S but not in fraction B (Figure 1b). Dynamic light scattering (DLS) measurement revealed differences in EV profiles between the isolation methods and detected the presence of ‘particles’, most likely protein aggregates, in fractions B (Figure 1c). The quality of the EV purification in both pipelines was also confirmed by Western blotting (WB) of four EV‐associated proteins (Flotillin1, Flotillin2, CD9 and CD81) (Figure 1d). Of note, differences in the signal intensity between fractions U and S were caused by approximately 15 times higher input material in fractions U than in fractions S (Figure 1a, e). Lipoprotein particle marker ApoA‐1 was present in all three fractions, which agrees with an earlier report showing that neither EV isolation method can completely deplete lipoproteins (Karimi et al., 2018). However, ApoA‐1 was significantly more abundant in fractions B, indicating that both EV isolation methods can considerably reduce the amount of lipoprotein particles in this type of biological sample.

**FIGURE 2:**
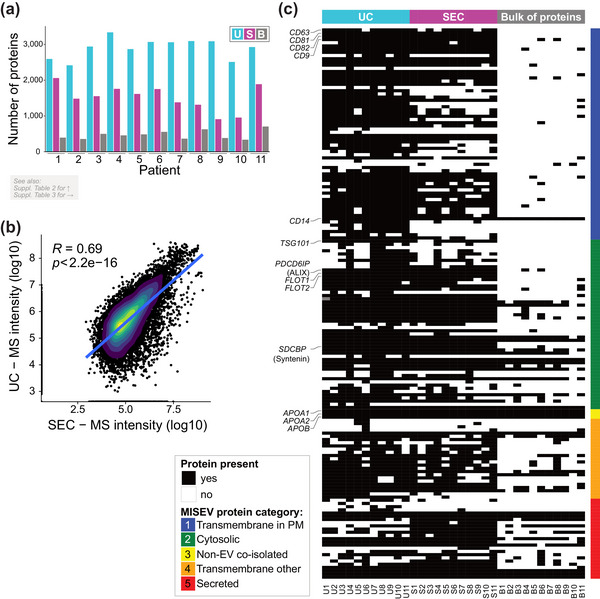
Characterization of isolated EV isolates by tandem mass spectrometry. (a) Barplot showing number of proteins identified by mass spectrometry in each fraction for individual patients. (b) The Pearson's correlation of log transformed data of all protein intensities detected by MS in EVs isolated by UC and SEC in patients 1–11. Blue line represents the linear regression. (c) Heatmap depicting overlay of mass spectrometry data with the list of EV‐associated proteins (five categories) published in MISEV2018 (Théry et al., 2018). U = EVs isolated by ultracentrifugation, S = EVs isolated by size‐exclusion chromatography, B = bulk of non‐EV proteins, isolated by size exclusion chromatography.

Following the sample validation described above, we have submitted all 33 samples (U1‐U11, S1‐11, B1‐11) to quantitative label‐free MS/MS analysis. The list of proteins identified in the individual samples is available in Table S2 and summarized in Figure 2a. More proteins could be identified in U fractions (2415–3093) as compared to S (909–2060) and B fractions (335–704). The difference in proteins identified likely reflects the higher protein amount in the EV‐containing sample obtained by UC (compare U vs. S, see also Figure 1d) and high abundance but low diversity in the B fractions (see also amido black‐stained membrane in Figure 1e). The Pearson's correlation coefficient between all protein intensities detected in U and S fractions was moderate—R = 0.69 (Figure 2b). In addition, we utilized a recent proteomic study (Lischnig et al., 2022) that identified proteins enriched in large versus small EVs as a reference for comparing individual fractions (Figure S1c). S fractions demonstrated only a modestly increased proportion of proteins enriched in large EVs in comparison to the U and B fractions (Figure S1c’).

As the first step in the analysis, we compared our data with the list of EV‐associated proteins published in the *minimal information for studies of extracellular vesicles* (MISEV2018) (Théry et al., 2018). Of 390 proteins reported in this reference, 172 were detected in at least one measured sample (Figure 2c, Table S3). The vast majority of these proteins belong to category 1, ‘Transmembrane or GPI‐anchored proteins associated to the plasma membrane and/or endosomes’, or category 2, ‘Cytosolic proteins recovered in EVs’; these proteins were present primarily in U and S fractions, but largely absent in B fractions, suggesting that our EVs isolations were of high quality.

**FIGURE 3:**
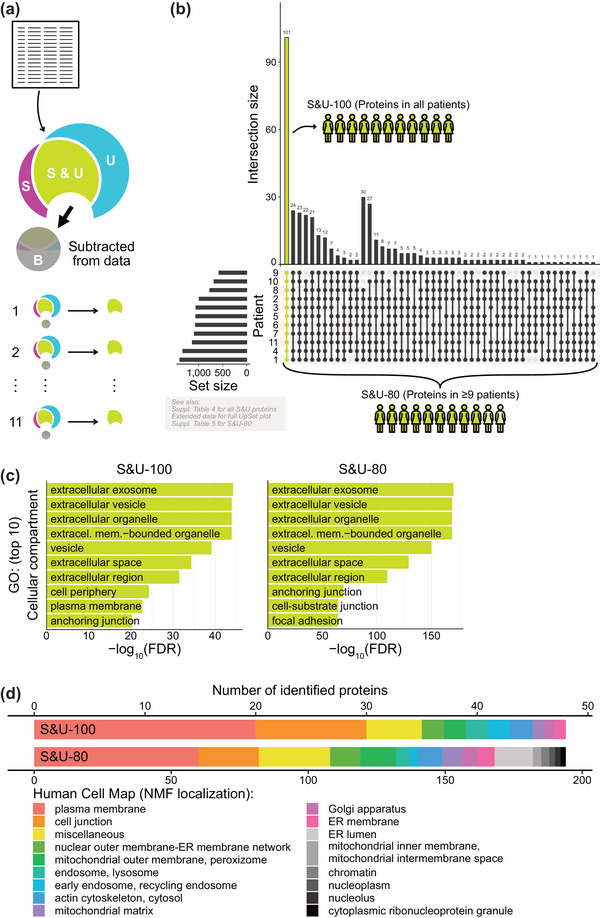
Identification and characterization of core ascitic EV‐associated proteins. (a) Schematic overview of processing of data obtained from mass spectrometry analysis. Data were processed in a patient‐dependent manner. S = proteins from EVs isolated by size‐exclusion chromatography, U = proteins from EVs isolated by ultracentrifugation, S&U = protein is present in both S and U, B = bulk of non‐EV proteins isolated by size exclusion chromatography, that are subtracted from S&U. Only data from S&U (lime green) are further analyzed through this figure. (b) UpSet plot visualizing intersections of sets of proteins between patients. Only sets present in ≥9 patients are shown (S&U‐80). One hundred one of these proteins are present in all 11 patients (S&U‐100). (c) Gene ontology analysis for the top 10 cellular compartments of S&U‐100 and S&U‐80 identified by both methods. FDR—false discovery rate. (d) Stacked barplots showing cellular localization for S&U‐100 and S&U‐80. The analysis is based on results from Human Cell Map project (Go et al., 2021). NMF, non‐negative matrix factorization. Approximately half of the proteins from both groups were not identified in this database.

In order to identify the protein composition of EVs from HGSC‐associated ascites, we merged the data from two purification pipelines, essentially as outlined in Figure 3a. Each isolation method has limitations and inclination towards method‐specific contaminants. As such, we next considered only proteins identified by UC and SEC (S&U, lime green) for each patient and not present in the corresponding B fractions as actual EV proteins. The number of EV proteins in S&U ranged between 580 and 1392 per patient and was mostly limited by the number of proteins isolated from S fractions (Figure S1a, a’, Extended data, Table S4).

As expected, there was a pronounced individual variability among patients. However, there are still many S&U proteins found in the majority or all patients (Figure 3b). We consider these proteins to be ‘core ascitic EV‐associated proteins.’ For future analysis, we set up two categories of stringency—100% (i.e., found in all patients; S&U‐100) and more than 80% (found in ≥9 out of 11 patients; S&U‐80). In the S&U‐100 group, we detected 101 core ascitic EV‐associated proteins (Table 1); in the S&U‐80 group, 392 proteins found in ≥9 patients (Table S5). In total, we found 18 proteins (17.82%) of the S&U‐100 and 50 proteins (12.75%) from S&U‐80 in the MISEV2018 list of EV‐associated proteins (Théry et al., 2018). These proteins involve classical EV markers such as CD81, CD82, Flot1 and Flot2. Gene ontology (GO) analysis of S&U‐100/80 proteins showed extreme enrichment of GO categories associated with EVs, such as extracellular exosome and extracellular vesicle (Figures 3c and S1b), suggesting that these proteins are truly integral EV proteins. Next, we performed analysis of overrepresented biological pathways using Reactome database (Gillespie et al., 2022). Among the most relevant top‐level pathways for both S&U‐100 and S&U‐80 groups, are Immune system, Hemostasis, Signal Transduction, Neuronal system, Disease, Transport of Small molecules, Cell‐Cell communication and Extracellular matrix organization (Figure S2a,b), the 25 most relevant pathways for each group are summarized in Figure S2c,d. To characterize the groups of proteins further, we took advantage of the Human Cell Map, which defined the subcellular localization of human proteome using unbiased proteomics (Go et al., 2021). Based on this, most of the core ascitic EV‐associated proteins in both groups were localized subcellularly in the plasma membrane or cell junction (Figure 3d). About half of the core ascitic EV‐associated proteins were identified using this approach. The low recovery of cell identification suggests diverse sources of EVs from ascites of HGSC patients, which contained multiple cell type‐specific proteins that are not expressed in HEK293, the cell line used as a basis in the Human Cell Map. Altogether, we identified a set of ‘core ascitic EV‐associated proteins’ encompassing 392 items. This set contains typical EV markers, lacks proteins routinely contaminating EV isolates, and broadly covers interpatient heterogeneity.

**TABLE 1:** One hundred one core ascitic EV‐associated proteins identified in all patients.

Secretedextracellular	Membraneous	Cytosolic	Multiple or unknown locations
FCN1	ANO6	ABI1	ADD1
PLEKHO2	AP2A1	ARHGDIB	AHCYL1
TNFAIP2	AP2B1	ATP5B	ANXA7
	ATP1B3	CAB39	ARF6
	ATP2B1	CCT3	BST2
	BSG	CNDP2	CAPZA2
	BST1	EPS8L1	CD59
	CD151	GSTO1	CLIC5
	CD81	LTA4H	CRIP2
	CD82	PCMT1	CYFIP1
	CLDN1	RPL8	DNAJC5
	F11R	RPS26	EGFR
	FAM49B	RPS7	EPB41
	FLOT1	S100A16	EPB41L2
	GNAQ	STXBP3	EPB41L3
	GPRC5C	ZC3HAV1	FAM129B
	HLA‐C		FLOT2
	HLA‐DPA1		FOLR2
	HLA‐DRB3		GNA11
	ITGAM		GNA13
	ITGB1		GNAI1
	ITGB2		GNAI3
	MRC1		GNAS
	MYADM		HCK
	NCKAP1		HLA‐DRB5
	PECAM1		ILK
	PTGFRN		IPO5
	PTPRJ		KRAS
	RAP2B		LYN
	RHOG		PARK7
	RP2		PLSCR1
	RRAS2		PLSCR3
	SLC16A1		RAB8A
	SLC16A3		RAC1
	SLC1A5		RDX
	SLC34A2		RFTN1
	STK10		RHOA
	TGM1		SLC9A3R1
	THY1		SMPDL3B
	VAMP8		STOM
			STXBP2
			TMSB4X

*Note*: The cellular localization was assigned according to data in UniProt database.

### 3.2 Identification of ascitic EV proteins unique for HGSC

**FIGURE 4:**
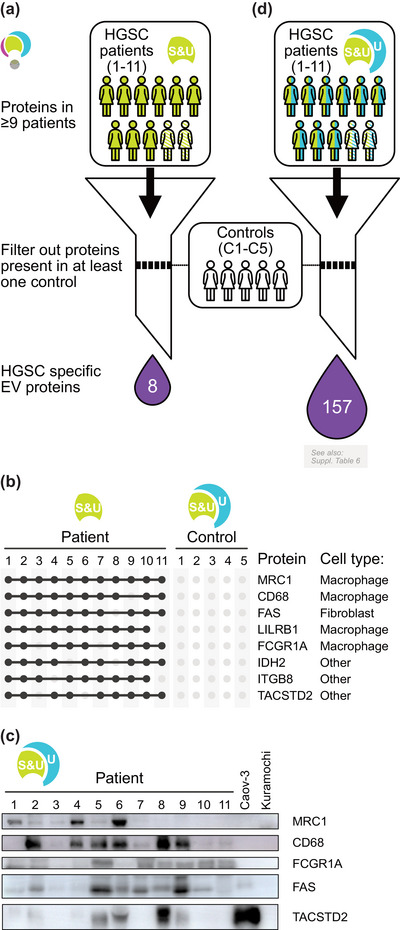
Identification of ‘HGSC‐specific EV‐associated proteins’. S = proteins from EVs isolated by size‐exclusion chromatography, U = proteins from EVs isolated by ultracentrifugation, S&U = protein is present in both S and U. (a) Schematic overview of identification of eight HGSC‐specific EV‐associated proteins. (b) Presence of eight HGSC‐specific EV‐associated proteins among patient samples. (c) Western blotting for selected HGSC‐specific EV‐associated proteins on U fractions of 11 patients and Caov‐3 and Kuramochi HGSC cell lines. Caov‐3 and Kuramochi cells are used as negative controls for macrophage markers MRC1, CD68, and FCGR1A. For TACSTD2, Caov‐3 serves as a positive control, while Kuramochi serves as a negative control. (d) Schematic overview of identification of 157 HGSC‐specific EV‐associated proteins. These data were augmented by proteins identified by UC.

Ascites of HGSC patients contain a mixture of cells, proteins, and EVs present in the homeostatic peritoneal fluid and those that are a result from tumour growth. We expanded our cohort with 5 control EV samples from non‐malignant peritoneal effusions isolated by UC to distinguish between these two categories. To find HGSC‐specific EV‐associated proteins, we have subtracted each ‘core ascitic EV‐associated protein’ (see Figure 3, S&U‐100/80) that was detected in any of the control samples (Figure 4a). This stringent filter removed a great majority of ascitic EV proteins except for eight proteins uniquely concomitant with the ascites from HGSC patients. Interestingly, annotating these HGSC‐specific EV‐associated proteins suggested that they may originate from various cell types and not just malignant cells. Unbiased annotation using the CellKb, a reference database of cell type marker genes (Patil & Patil, 2022), suggested that four identified proteins were reported as markers of macrophages (MRC1 alias CD206, CD68, LILRB1, FCGR1A), one as a marker of fibroblasts (FAS) and three others (IDH2, IGB8, TACSTD2) were not directly connected with a specific cell type (Figure 4b). To validate the specificity of these identifications, we performed western blotting on fractions U obtained from both patients and control cell lines, targeting five of these markers as illustrated in Figure 4c.

The observation that many or even majority of HGSC‐specific ascitic EV‐associated proteins do not originate from tumour cells attracted our attention. In the next step, we re‐analyzed our input data by changing the core ascitic EV‐associated proteins from proteins found in ≥9 patients in S&U to proteins found in ≥9 patients in U samples (after removing B fractions). These present a significant portion of all identified proteins (Figure S1a) and thus may contain important hits that would have been otherwise missed. As shown in Figure 1d,e, UC concentrates EVs and allows for the detection of less abundant proteins. To make sure that the considered proteins originate from the HGSC‐associated ascitic EVs, we have applied three criteria: (i) protein not detected in the same patient in fraction B; (ii) protein detected at least in one sample obtained by SEC, an independent method for EV isolation (S1‐S11) and (iii) protein absent in all EV isolations from control non‐malignant fluids. This analytical pipeline (Figure 4d) added another 149 proteins (Table S6) to the list of eight proteins identified in Figure 4a. We propose that these 157 proteins represent EV‐associated proteins specific for pathological ascites present in HGSC. We believe this list contains both important players of HGSC progression and potential disease biomarkers. Interestingly, the analysis of over‐represented biological pathways using Reactome database revealed, that in contrary to numerous relevant top‐level pathways for both S&U‐100 and S&U‐80 groups (Figure S2), there are only two, namely Immune system and Protein localization, overrepresented among these HGSC‐specific proteins (Figure S3a); with the Immunoregulatory interactions between a Lymphoid and a non‐Lymphoid cell being the most significant pathway (Figure S3b).

### 3.3 Cellular origin of EVs in HGSC ascites

**FIGURE 5:**
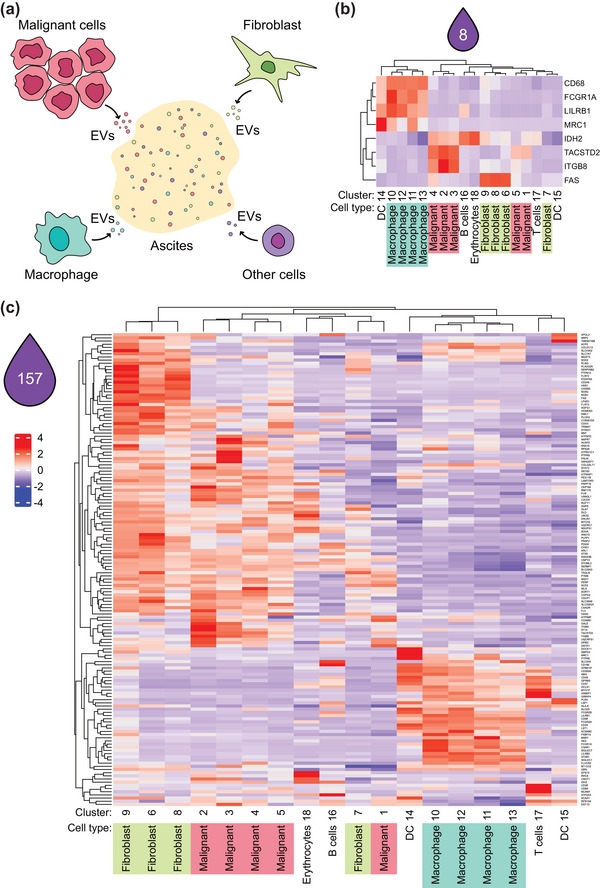
Cells of origin of EVs. (a) Scheme depicting various origins of EVs in ascites. (b, c) Heatmaps visualizing expression of genes of interest. Data and clusters are based on analysis by (Izar et al., 2020). (Gene names in heatmap C are legible upon zoom in).

Similarly to other biofluids, ascites likely contains EVs from various cell types. As EVs represent molecular fingerprints of the cells that produce them, we have attempted to identify to what extent different cell types contribute to the ascitic pool of EVs in HGSC (Figure 5a). To address this question, we took advantage of a recent study which profiled cells from HGSC patients by scRNA seq and annotated 18 different cell types/subtypes present in HGSC ascites (Izar et al., 2020). We have plotted the expression of the HGSC ascites‐specific EV markers (Figure 4) on the expression profiles of individual cell types from Izar et al. (2020) (Figure 5b,c). The eight stringent markers (Figure 4b) showed a cell type‐restricted expression pattern: CD68, FCGR1A, LILRB1 and MRC1 are expressed in the ascitic milieu only by macrophages, TACSTD2 (also known as TROP‐2) and ITGB8 by malignant carcinoma cells, FAS by fibroblasts and IDH2 predominantly by B‐lymphocytes and erythrocytes. We have observed a very similar trend on the more extensive set of 157 EV markers (Figure 4d), where many of the markers were expressed almost uniquely by one of the dominant cell types (macrophages, fibroblast, malignant cells and lymphocytes) (Figure 5c). This data strongly suggests that individual EVs specific for malignant ascites originate from different cell types and provides a proof‐of‐concept that the analysis of EVs can be used to assess the composition of TME.

**FIGURE 6:**
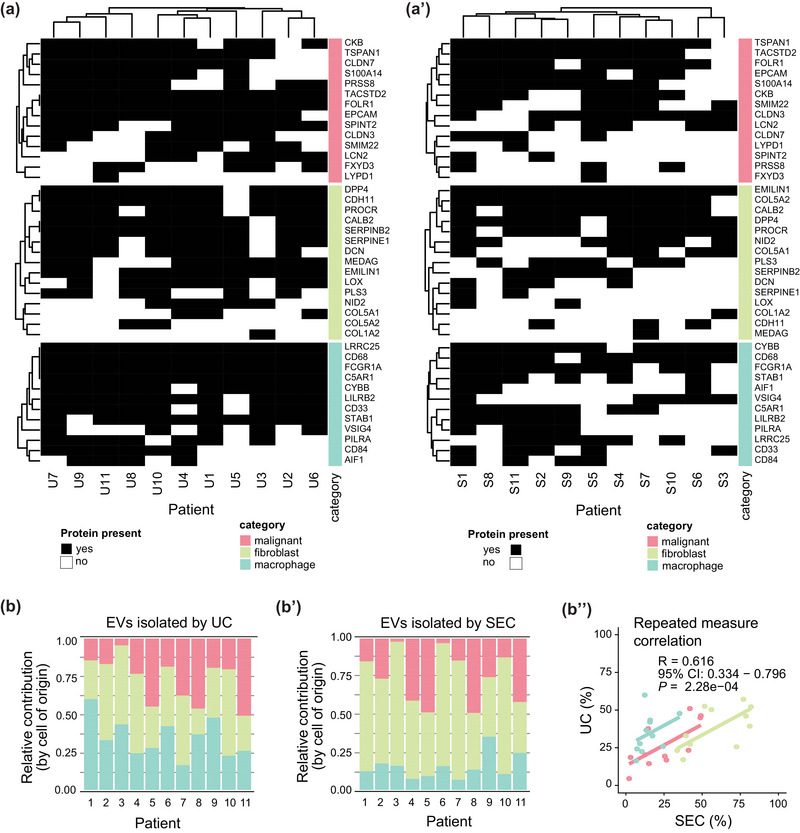
Analyses of ascitic EVs by cell of origin. (a) Heatmap visualizing presence of the cell type‐specific markers, by (Izar et al., 2020) found in our mass spectrometry data of EVs samples isolated by UC (a) and SEC (a’). (b) Graph showing proportions of cell types according to protein intensities of cell type‐specific markers for each patient. Data are based on protein intensities measured on EVs samples isolated by UC (b) and SEC (b’). (b’’) shows correlation of the (b) and (b’) data. CI, confidence interval.

The varying composition of EVs in each patient allows the use EVs as markers describing the cellular diversity of TME in HGSC. To explore this potential, we have designed a panel of markers detected in our EV preparations by both UC and SEC and, at the same time, are cell‐type specific. To provide insight into the TME, we restricted our analysis to the protein in the HGSC‐associated ascites produced only by one of the most common cell types—that is, macrophages, fibroblasts and malignant (epithelial) cells. Forty‐five markers representing these categories, according to Izar et al. (2020), were selected (details in Section 2). Moreover, their presence in each patient EV samples were scored for samples obtained by UC (Figure 6a) and by SEC (Figure 6a’). Having the ability to predict the cell type of origin for a particular EV protein, we quantified the contribution of macrophages, tumour cells, and fibroblasts to the EV proteins in each patient. This analysis has been performed for samples obtained by both UC (Figure 6b) and SEC (Figure 6b’). Despite some variation between UC and SEC samples, the relative enrichment of macrophage markers in UC and fibroblast markers in SEC, the global patterns were comparable, and the results obtained by both methods correlated, R = 0.616 (Figure 6b’’). Independently of the method used for EV isolation, these results show significant inter‐patient variability and confirm our previous assumption that the majority of EV proteins detected in ascites across patients originate from cells of the TME (fibroblasts and macrophages) and not from malignant cells (Figure 6b).

### 3.4 The cellular composition of ascites does not correlate with its cell type‐specific EV content

**FIGURE 7:**
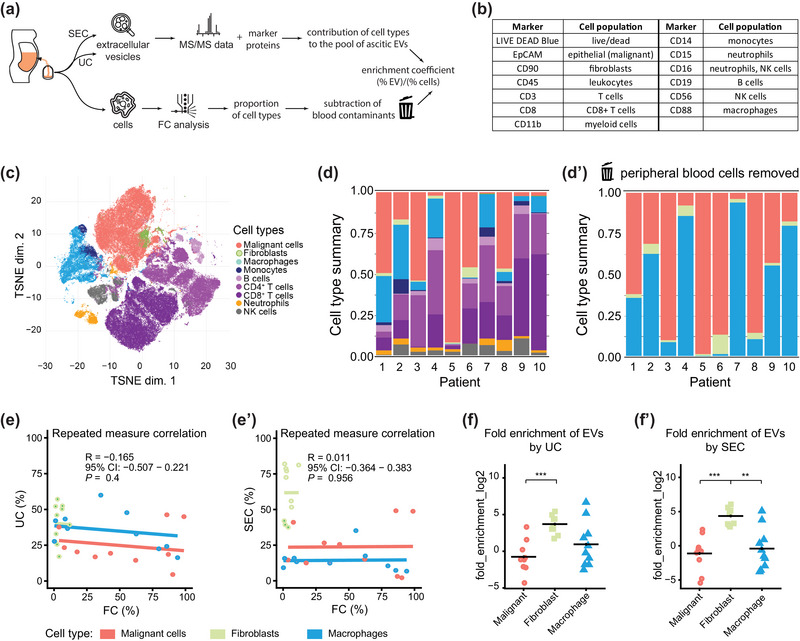
Analyses of ascitic cells. (a) Scheme of the analysis of ascitic cells and EVs. (b) Table of markers used for FC. (c) TSNE plot of markers detected by spectral flow cytometry on all ascitic cells. (d) Graph showing proportions of cell types detected by FC in ascites of individual patients using FlowSOM clustering. (d’) shows proportions of malignant cells, fibroblasts and macrophages detected by FC in ascites of individual patients after removal of populations of peripheral blood cells (possible contaminants). (e) The repeated measure correlation between proportions of major cell populations derived from FC data and from measurements of EV proteins. Data are based on protein intensities measured on EVs samples isolated by UC (e) and SEC (e’). CI—confidence interval. (f) Graphs showing ‘enrichment’ of EVs versus cells in individual ascites. The enrichment coefficient (EC) is defined as fold enrichment of (% EV)/(% cells). Proportions of cell types based on protein intensities measured on EVs samples isolated by UC (f) and SEC (f’) were plotted against proportions of major cell types (according to Izar et al., 2020) detected by FC.

Our data demonstrate that several key cell types known to form the HGSC microenvironment produce EVs to the malignant ascites. We show that there is a remarkable variability among patients. However, it is unclear how the EV composition of ascites reflects its cellular composition and whether it can help stratify HGSC patients. We have directly analyzed the ascitic cells to address this issue using flow cytometry (FC). Cells isolated from ascites at collection (frozen in 10% DMSO) were available for 10 of 11 patients (patients 1–10). We subjected them to a multiparameter analysis by spectral FC (Figure 7a) (details in Section 2 and Table S7). The panel of markers (Figure 7b) allowed us to identify malignant cells, fibroblasts, macrophages and multiple cell types from peripheral blood (B cells, CD4^+^ and CD8^+^ T cells, neutrophils, monocytes and NK cells). FlowSOM has performed the cell type clustering and annotation in each patient. The tSNE plot of markers used is depicted in Figure 7c. Populations identified by FlowSOM were subsequently verified on non‐transformed data using a manual gating strategy (Figure S4a). FlowSOM analysis data correlated very well with the manual gating, R = 0.95 (Figure S4b,b’).

The ratio of individual cell types in ascites revealed the heterogeneity of ascitic cells in the individual patients (Figure 7d). In many cases, lymphocytes formed the prominent cell population, contrasting with the low abundance of lymphocyte‐specific proteins in the EV samples. This led us to speculate that lymphocytes may originate from bleeding into ascites, which occurs naturally during the debulking surgery. Therefore, we calculated the proportion of cells after removing the cell populations commonly found in the peripheral blood, that is, lymphocytes, NK cells, monocytes and neutrophils. As a result, we quantified only three significant cell populations in the ascites according to Izar et al. (2020), that is, malignant cells, fibroblasts and macrophages (Figure 7d’).

Interestingly, in our patient cohort, we have not observed any correlation between the abundance of macrophages, fibroblasts and tumour cells assessed by FC (Figure 7d’) and the abundance of EV proteins specific for these cell types (Figure 6b). This has applied both for the EVs isolated by UC (Figure 7e; R = −0.165) and for EVs isolated by SEC (Figure 7e’; R = 0.011).

However, when we compared the proportion of cell populations measured by FC and derived from the EV protein composition using enrichment coefficient (EC = % of cell type‐specific EV markers divided by % of cells), EC was consistent for each cell type across patients, irrespective of the EV isolation method (Figure 7f, by UC; Figure 7f’, by SEC). We have observed that fibroblast‐derived EV proteins were notably enriched—EC mean 17.3 (UC) and 27.1 (SEC), in comparison to malignant cells (mean 1.66 by UC and 1.26 by SEC), while in macrophages, the mean was 15.1 (UC) and 5.22 (SEC). Together, these results demonstrate that analysis of cell type‐specific EV proteins provides extra value that complements the analysis of the cellular composition of ascites. EVs in the ascites probably integrate complex information from the tumour and the TME from the cells floating in the ascites and the solid tissue in contact with the peritoneum.

### 3.5 Cell type‐specific EV‐associated proteins predict survival of HGSC patients

**FIGURE 8:**
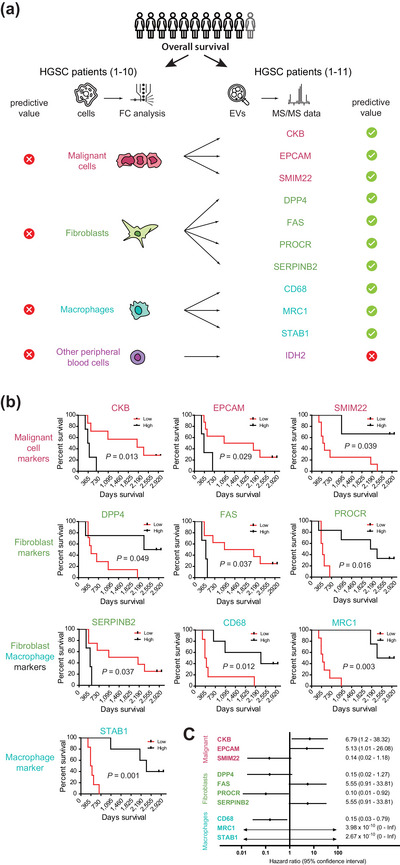
Survival analyses. (a) Scheme of the analysis which cellular and EV parameters in ascites correlate with overall survival of HGSC patients in our study. (b) Overall survival of patients based on the composition of ascitic EVs. Log‐rank (Mantel‐Cox) Test on protein intensities in U samples. (c) Forest plot of OS analysis in b). HR, hazard ratio. Extended data: UpSet plot visualizing all intersections of sets of proteins identified in EVs isolated by both methods. Each of 2418 proteins in this graph was recovered after both SEC and UC simultaneously in at least one patient. Intersections highlighted in lime green consist of sets of proteins found in ≥9 patients.

The analyses shown in the previous chapter suggest that two critical components of ascites—ascitic cells and ascitic EVs—do not provide overlapping information concerning the biological and possibly the clinical behavior of the tumour and TME. Therefore, we addressed whether any diagnostic readouts—cellular or EV composition of the ascites—have a prognostic value. We investigated the effect of the composition of the ascitic milieu on patient outcomes using overall survival (OS). Specifically, we tested whether the proportion of individual cell types (malignant cells, fibroblasts, macrophages and peripheral blood cells) in ascites associated with the OS of analyzed patients (details in Table S1); however, none of the cellular parameters correlated with patient survival (Figure 8a, left, Table S8). Next, we analyzed EV markers specific for these cell types (depicted in Figure 6a) and four additional EV‐associated proteins specific for HGSC, which we found to have cell type‐restricted expression patterns (FAS, IDH2, ITGB8 and MRC1, Figure 5b) and their capacity to predict the OS (details on the analysis can be found in Section 2 and complete results in Table S8). Intriguingly, out of 45 cell type‐specific proteins, 10 of them were potentially discriminative (Figure 8a, right). We have observed a strong positive effect on the OS in the case of the core macrophage markers CD68, MRC1 and STAB1 a malignant cell marker SMIM22 and fibroblast markers DPP4 and PROCR. On the contrary, we have observed a strong negative effect on the OS in the case of markers, which are expressed in ascitic milieu only by malignant cells (EPCAM and CKB) and fibroblasts (FAS and SERPINB2) (Figure 8b,c).

We conclude that our data provides evidence that the analysis of EV‐associated proteins from the ascites of HGSC patients is suitable as a complex footprint of the functional state of the tumour and its microenvironment. As such, it holds an enormous potential that can be exploited to improve our understanding of tumour biology, streamline diagnostics, and guide the therapy of this deadly disease.

## 4 DISCUSSION

Malignant ascites is a complex biofluid contributing to the formation of metastases and the development of chemoresistance of HGSC. It is a unique source of various cell types, soluble factors, and EVs that resemble those in the blood (Holcar et al., 2021; Kipps et al., 2013). It has been recently shown that EVs found in malignant ascites can also be found in the plasma of these patients (Wang et al., 2022). However, ascites has several advantages in comparison to plasma. Most notably, ascites is directly in contact with the tumour and its environment, leading to considerably higher concentrations of tumour‐derived factors in ascites than plasma. Moreover, ascites is often present in large volumes (Ford et al., 2020; Kipps et al., 2013), providing more input material and enabling us to detect highly abundant EVs present in the sample and rare EVs.

Despite recent advances in the field, the isolation of pure EVs from complex fluids (like blood and ascites), where proteins and lipoproteins vastly outnumber EVs, remains impossible (Dong et al., 2020; Holcar et al., 2021; Karimi et al., 2018). EV isolation methods are not equal in terms of purity of EVs; some enrich only subpopulations of EVs. Therefore, it is crucial to select a method suitable for downstream analyses. In this study, we exploited the relatively large amount of patient ascites and isolated EVs by SEC and UC, the two most used methods for EV isolation. SEC separates the size of the particles as they flow through the stationary phase, with larger particles eluting first. SEC efficiently removes soluble proteins and high‐density lipoproteins but usually co‐isolates lipoproteins with a similar size as EVs (Askeland et al., 2020; Holcar et al., 2021; Ter‐Ovanesyan et al., 2021). UC separates particles based on their sedimentation coefficient (reflecting hydrodynamic size and density). UC reduces protein aggregates and lipoproteins with different densities than EVs (notably when using the flotation step), but soluble proteins and lipoproteins are still co‐isolated (Brennan et al., 2020; Holcar et al., 2021). Several studies report that SEC has a higher yield of EVs but worse purity and overall protein contaminants than UC (Takov et al., 2019; Tian et al., 2020).

Our study provides matching data from both UC and SEC, which allows the possibility to compare the biases of each method separately. Similarly, others showed on EVs from the human plasma that parallel isolation by SEC and UC results in different populations of EVs (Askeland et al., 2020; Tian et al., 2020). Our data showed that some UC samples still had protein contamination (Figure 1b,c) even though we used a sucrose cushion flotation step to reduce these contaminants. qEV Original (70 nm) used for SEC isolation has an optimal recovery rate of 70–1000 nm, which is reflected as polydisperse samples in Figure 1c. Despite this polydispersity, our analysis of proteins specifically enriched in large and small EVs revealed no significant disparity in their abundance between the U and S fractions (Figure S1c,c’). Of important note, our approach differs from the method used in the reference study by Lischnig et al., where large EVs were enriched by centrifugation at 16,500 × *g* and small EVs by centrifugation at 118,000 × *g*. Moreover, a substantial number of EV proteins in our dataset were not identified in the reference study, possibly attributed to their distinct origin as derivatives of the MDA‐MB‐231 breast cancer cell line (Lischnig et al., 2022). Therefore, the limitation of our study is that by considering only proteins present in both isolation methods, we might miss proteins from larger vesicles that might be isolated by only SEC. On the other hand, combining both methods allowed us to identify the subset that we call ‘core ascitic EV‐associated proteins’, where the method‐introduced bias was eliminated.

SEC using qEV columns is closest to the clinical application among the currently available EV isolation methods. Of significant note, the ‘core ascitic EV‐associated proteins’, which we identified, are detectable after the isolation of EVs by SEC from a complex biofluid and thus can be utilized for biomarker discovery in the field of HGSC.

In our quest to identify potential biomarkers for HGSC, the selection of an appropriate negative control becomes paramount. To comprehensively address this question, we integrated both distinct inflammatory controls, represented by non‐malignant ascites from ovarian hyperstimulation syndrome and cystic fluid, as well as non‐inflammatory controls, the follicular fluids (refer to Table S1 and Section 2 for details). This strategy aimed to encompass diverse controls in our proof‐of‐principle study, enabling us to differentiate disease‐specific proteins while considering the intricate interplay between inflammatory and non‐inflammatory conditions. However, it is noteworthy that a significant age discrepancy exists between the patients (median age 63 years) and controls obtained from IVF clinics (median age 30 years) due to prevailing ethical approvals. Recognizing the significance of accounting for age differences, particularly in light of the potential influence of age on EV composition (Noren Hooten et al., 2022), we commit to addressing this concern in future studies. Our forthcoming investigations will include better age‐matched controls, such as peritoneal lavages from women who underwent surgery for uterine/vaginal descent.

Our exploration for EV‐associated markers specific to HGSC, conducted under stringent criteria, revealed eight proteins. Notably, TACSTD2 (also known as TROP‐2) recently emerged as a HGSC‐specific small EV marker, alongside FRα and Claudin‐3, employing a microfluidic device for EV isolation, specifically polyketone‐coated nanowires (Yokoi et al., 2023). ITGB8 (Integrin beta‐8), previously identified as an independent predictor of unfavorable survival in HGSC (He et al., 2018), demonstrated an association with cisplatin resistance (Cui et al., 2018), indicating our data are in line with prior research. The expression of CD68, FCGR1A, LILRB1 and MRC1 in the ascitic milieu by macrophages and dendritic cells (DC) underscores the significant involvement of immune cells. Furthermore, the pathway analysis of an expanded list of HGSC‐specific EV‐associated markers (Figure S3) emphasized the participation of these proteins in immunoregulatory interactions between lymphoid and non‐lymphoid cells, as well as the citric acid (TCA) cycle and respiratory electron transport.

Like other cancers, the OC TME consists of tumour, immune, and stromal cells. While some immune and stromal cells promote cancer progression and metastasis, some suppress it. Ascites is in direct contact with these cells (many of them float directly in the ascites) and mediate the transport of EVs between them (Ford et al., 2020; Kipps et al., 2013; Ritch & Telleria, 2022). Publications reporting EVs from OC ascites usually search for biomarkers or study how ascitic EV regulate various pro‐tumourigenic and pro‐metastatic features of cancer cells (Bortot et al., 2021; Cai et al., 2021; Li et al., 2019; Mitra et al., 2021; Wang et al., 2022). In our study, we combined the proteomic data with the single‐cell analyses to identify the sources of EVs in the ascites. We also demonstrated that many, if not most, EV proteins are cell‐type specific. We are aware of only one study that attempted to determine the cell of the origin of EVs of cancer patients from biofluids using proteomics. Hoshino et al. uncovered the patient's cancer type by proteomic profile of EVs isolated from plasma (Hoshino et al., 2020). To our knowledge, we have novelly attributed markers to various subpopulations of EVs originating from different cell types that form tumours and their microenvironment. Moreover, we describe the remarkable heterogeneity of ascitic EVs among patients, in line with the described heterogeneity of ascitic cells (Izar et al., 2020; Kipps et al., 2013).

As previously mentioned, ascites contains multiple cell types. In addition to tumour cells, the most common are macrophages and fibroblasts (Izar et al., 2020; Kipps et al., 2013). Both macrophages and fibroblasts impact virtually all aspects of disease progression and communicate profoundly bi‐directionally with tumour cells (Rickard et al., 2021; Ritch & Telleria, 2022). Indeed, malignant cells, macrophages and fibroblasts were the most prominent sources of EVs in our samples, which suggests that EVs play a significant role in communication between these cells. The direct analysis of the cellular content of ascites has confirmed that these cell types were present in the ascites from which EVs were isolated. Fibroblasts, one of the most prominent sources of EVs, were present scarcely in ascites and were underrepresented compared to the fibroblast‐specific markers carried by EVs. This is either suggestive of their high EV secretion capacity or can illustrate that fibroblasts, as typically adherent cells, do not float in ascites. Instead, fibroblasts are located in tissues in contact with ascitic fluid and are abundant in the peritoneum, especially in proximity to the primary tumour and metastatic sites where they participate in abundant communication with tumour cells (Rakina et al., 2022; Ritch & Telleria, 2022). Please note that we use the term ‘fibroblasts’ in accordance with the terminology established by Izar et al. In their study, four distinct subpopulations of fibroblasts were annotated, all of which are identified as cancer‐associated fibroblasts (CAFs). Therefore, in our investigation, when we refer to ‘fibroblasts’, we specifically mean cancer‐associated fibroblasts (CAFs).

It remains an open question: What is the significance of the ascitic lymphocytes and other cell types primarily in the peripheral blood? Lymphocytes were reported to be the second most common cell type reported in ascites (Kipps et al., 2013) and are believed to be the receivers for EVs from tumour cells, macrophages and fibroblasts. In contrast, EV‐associated markers of lymphocytes from the ascites are underrepresented when compared to the proportion of lymphocytes in the ascites. Accordingly, lymphocyte signaling through EVs towards tumour cells, macrophages and fibroblasts is negligible (Lucotti et al., 2022). However, in light of our findings, this interpretation becomes complicated. Our results suggest lymphocytes and other peripheral blood components can represent the contamination of ascitic fluid during bleeding associated with the collection of ascites.

The evaluation of the clinical potential of EV‐associated and cell type‐specific markers pointed to the critical contribution of macrophages to the ascitic TME. In the context of macrophages, EVs are usually studied as the particles that are up‐taken by macrophages, but very few studies focused on macrophage‐derived EVs. It is striking that in our patient cohort, the macrophage‐specific EV‐associated markers, namely MRC1, CD68 and STAB1, but not the proportion of macrophages in ascites, predicted survival in HGSC patients. This suggests that the analysis of the markers carried by EVs integrates the information on the activity and number of macrophages from both the ascites and surrounding solid tissue. Together, these results reveal an exciting avenue for future diagnostics and treatment of HGSC.

Our results suggest that macrophages and/or their EVs have protective effects in HGSC and limit tumour progression. While CD68 is often used as a marker for macrophages and STAB1 (Stabilin‐1) encodes a scavenger receptor, MRC1 (Mannose Receptor C‐Type 1, or CD206) plays a crucial role in recognizing and clearing glycoproteins. MRC1 was identified as one of the signature genes of tissue‐resident macrophages (RTMs) present in various human tissues (Chakarov et al., 2019). Recent research has underscored the significance of RTMs as a notable source of macrophages within ascitic fluid and identified that macrophages of different origins and phenotypes coexisted within the ascites (Zheng et al., 2023). We explored also additional potential biomarkers, identified in our study. EpCAM (Epithelial Cell Adhesion Molecule), a recognized marker of malignant cells, is considered indicative of EVs originating from malignant cells. Higher levels of EpCAM‐positive EVs are thus interpreted as reflecting increased tumour burden, and consequently, negatively associated with patient survival across various cancers. Notably, EpCAM is often utilized as one of markers for EVs for the early diagnosis of HGSC (Jo et al., 2023; Li et al., 2023). Similarly, CKB (Creatine Kinase B), expressed in malignant cells, exhibited a negative correlation with patient survival. This is consistent with previous reports indicating CKB up‐regulation in epithelial OC and its potential as a serum marker (Huddleston et al., 2005). Additionally, knockdown of creatine kinase B demonstrated inhibition of OC progression by reducing glycolysis (Li et al., 2013). While SMIM22 (Small Integral Membrane Protein 22) was found to be upregulated in OC tissues (Wei et al., 2022), our data showed a positive correlation between SMIM22 on EVs and patient outcomes. On contrary, FAS‐ and SERPINB2‐positive EVs exhibited a negative correlation with patient survival in our study. FAS (Tumour necrosis factor receptor superfamily member 6) and SERPINB2 (Serpin Family B Member 2, also known as Plasminogen activator inhibitor‐2) are expressed on various cell types but was reported by Izar et al. to be predominantly expressed by CAFs in HGSC ascitic milieu. Dysregulation of the FAS pathway, linked to resistance to apoptosis, could contribute to tumour development. DPP4 (Dipeptidyl Peptidase 4), also known as CD26, functions in immune system regulation and glucose level control. In OC, its expression was associated with increased chemosensitivity to paclitaxel (Kajiyama et al., 2010) and DPP4 inhibitor sitagliptin not only enhanced lymphocyte recruitment (Wilson et al., 2021) and prolonged survival in a syngeneic mouse model of OC but also modulated the response of OC cells to chemotherapeutic agents (Kosowska et al., 2020). Similarly, PROCR (Protein C Receptor), a cell surface receptor involved in the protein C anticoagulant pathway, displayed a positive association with patient survival in our study. PROCR, implicated in various biological processes such as inflammation and vascular biology, suggests potential implications for the tumour microenvironment and cancer progression.

In summary, we performed the first in‐depth MS/MS study of precisely isolated EVs from ascites of HGSC patients and identified HGSC‐specific EV‐associated proteins. We provide evidence that by combining of EV proteomics and single‐cell methods, we can assess the contribution of cells forming the tumour and its environment to the EV pool in the ascites. Surprisingly, analysis of cell type‐specific EV markers showed a more substantial predictive potential in HGSC than analysis of ascitic cells. Our study opens multiple directions for the follow‐up research that will address burning questions such as ‘Which cells produce EVs into the complex biofluids and how this dynamics changes with course of the disease and/or treatment?’ and ‘How can we use cell type‐specific EV markers for diagnosing and treating pathological conditions?’

## AUTHOR CONTRIBUTIONS

**Anna Vyhlídalová Kotrbová**: Formal analysis; investigation; methodology; validation; visualization; writing—original draft; writing—review and editing. **Kristína Gömöryová**: Data curation; formal analysis; methodology; validation; visualization; writing—review and editing. **Antónia Mikulová**: Data curation; formal analysis; investigation; methodology; visualization.**Hana Plešingerová**: Data curation; formal analysis; methodology; visualization; writing—review and editing. **Stanislava Sladeček**: Investigation. **Marek Kravec**: Investigation; writing—review and editing. **Šárka Hrachovinová**: Investigation. **David Potěšil**: Data curation; formal analysis; investigation; methodology; writing—review and editing. **Garett Dunsmore**: Data curation; formal analysis; methodology; validation; writing—review and editing. **Camille Blériot**: conceptualization; formal analysis; supervision. **Mathilde Bied**: Formal analysis; investigation. **Jan Kotouček**: Investigation; methodology. **Markéta Bednaříková**: Resources; supervision; validation. **Jitka Hausnerová**: Resources; supervision; validation. **Luboš Minář**: Resources; supervision. **Igor Crha**: Formal analysis; resources. **Michal Felsinger**: Investigation; resources. **Zbyněk Zdráhal**: Data curation; formal analysis; funding acquisition; methodology. **Florent Ginhoux**: Resources; supervision. **Vít Weinberger**: Resources; supervision. **Vitezslav Bryja**: Conceptualization; data curation; formal analysis; funding acquisition; methodology; supervision; writing—original draft; writing—review and editing. **Vendula Pospíchalová**: Conceptualization; data curation; formal analysis; funding acquisition; investigation; methodology; project administration; supervision; validation; visualization; writing—original draft; writing—review and editing.

## CONFLICT OF INTEREST STATEMENT

The authors report no conflict of interest.

## Supporting information

Supporting Information

Supporting Information

Supporting Information

Supporting Information

Supporting Information
